# Video-rate multimodal multiphoton imaging and three-dimensional characterization of cellular dynamics in wounded skin

**DOI:** 10.1142/s1793545820500078

**Published:** 2020-01-15

**Authors:** Joanne Li, Madison N. Wilson, Andrew J. Bower, Marina Marjanovic, Eric J. Chaney, Ronit Barkalifa, Stephen A. Boppart

**Affiliations:** * Department of Bioengineering, University of Illinois at Urbana-Champaign, Urbana, IL, U.S.A; † Department of Electrical and Computer Engineering, University of Illinois at Urbana-Champaign Urbana, IL, U.S.A; ‡ Beckman Institute for Advanced Science and Technology, University of Illinois at Urbana-Champaign, Urbana, IL, U.S.A; § Carle Illinois College of Medicine, University of Illinois at Urbana-Champaign, Urbana, IL, U.S.A

**Keywords:** Cellular dynamics, multimodal imaging, multiphoton microscopy

## Abstract

To date, numerous studies have been performed to elucidate the complex cellular dynamics in skin diseases, but few have attempted to characterize these cellular events under conditions similar to the native environment. To address this challenge, a three-dimensional (3D) multimodal analysis platform was developed for characterizing *in vivo* cellular dynamics in skin, which was then utilized to process *in vivo* wound healing data to demonstrate its applicability. Special attention is focused on *in vivo* biological parameters that are difficult to study with *ex vivo* analysis, including 3D cell tracking and techniques to connect biological information obtained from different imaging modalities. These results here open new possibilities for evaluating 3D cellular dynamics *in vivo*, and can potentially provide new tools for characterizing the skin microenvironment and pathologies in the future.

## 1. Introduction

The immune system is the most diffuse cellular system in the body, and all activities within this system are tightly regulated, including long-range cell migration and short-range communication by local chemical signaling or cell-to-cell contacts.^1^ Among the tools used to study this complex system, the introduction of multiphoton microscopy into the field of immunology has been proven to be incredibly powerful due to its ability to visualize time-lapse *in vivo* cell motility within native tissue environments.^1^ In addition, the use of a near-infrared wavelength excitation source also minimizes photodamage and photobleaching, and can achieve imaging at a deeper penetration depth. These characteristics are all ideal for long-term imaging deep into highly scattering tissue.^2^

Because multiphoton imaging can yield four-dimensional information (*x*, *y*, *z*, *t* of cell mobility and morphology, this wealth of data permits researchers to characterize a variety of parameters associated with cellular dynamics. However, the amount of data generated often produces far more information than can be digested by human observers. Therefore, reliable computational analysis tools present an opportunity to take full advantage of the available data in an efficient and reproducible manner. Numerous computational analysis platforms are available, including Matlab (Mathworks), ImageJ (NIH), and Cell Profiler,^3^ which are some of the well-known tools that allow researchers to characterize cell motility, including parameters such as mean velocity, displacement, and travel direction.^1,4–6^ Other features that can be quantified include cell morphology parameters, such as area, volume, and shape index.^4,5,7,8^ More advanced morphology measurements are possible through decomposing cell shape in terms of Zernike polynomials, or based on Fourier analysis, neural networks, or principle component analysis.^8–11^ The cell parameters and analytical techniques presented above have been widely utilized in characterizing not only cellular morphology, but also cellular activities.

Collagen fiber structure is another biological feature that has been heavily analyzed, most frequently after imaging using second harmonic generation (SHG) microscopy.^12,13^ As a tissue component that plays a key role in the pathological state of skin diseases and cancerous tumors, numerous computational methods have been developed to characterize its fiber structure.^12–16^ Some of the collagen parameters that have been investigated include fiber angle, length, width, density, and curvature. These parameters can also be characterized computationally using mathematical models, such as Fourier analysis,^14^ and various computer-assisted image feature extraction methods, including curvelet transform fiber extraction algorithm (CT-FIRE).^13,17,18^ Among other techniques, CT-FIRE is especially powerful because it integrates fiber-based preprocessing techniques with fiber extraction methods to improve fiber extraction accuracy, which leads to high sensitivity to changes in collagen alignment.^13^

To date, numerous computational analysis tools have been developed to quantify the multimodal cellular dynamics both at the molecular and cellular levels.^6,19–21^ However, few of the existing methods specifically focus on integrating multimodal information together in a temporally and spatially co-registered manner, primarily because of the limited number of multimodal optical imaging systems that are capable of generating these complex high-dimensional image datasets. In addition, the majority of these techniques focus exclusively on the cells in a relatively small field-of-view (FOV). While the information obtained from these methods provide useful molecular and cellular microenvironment information, these tools lack the ability to provide information from the macroenvironment where co-ordinated events among different tissue (skin) constituents can be better observed.

To address these challenges, a multimodal imaging analysis platform was developed, which was designed to analyze time-lapse multimodal imaging data acquired by a custom-built multimodal multiphoton microscope. This microscope integrates several imaging modalities commonly used to study the *in vivo* cellular environment, including two-photon excited fluorescence (TPF), SHG, and fluorescence lifetime imaging microscopy (FLIM). TPF is utilized for visualizing endogenous and exogenous fluorescence, SHG for collagen networks and organization, and FLIM for cellular metabolic activity.^12,22,23^ The imaging analysis platform developed in this study focuses on characterizing the three-dimensional (3D) datasets in a spatially and temporally co-registered manner, allowing users to quantify the relationship between different tissue constituents at any given time of an event. The applicability of the developed analysis platform was subsequently tested on a time-lapse 3D *in vivo* dataset acquired from wounded mouse skin.

## 2. Materials and Methods

### 2.1. 3D multimodal analysis platform

**Fig. 1.**
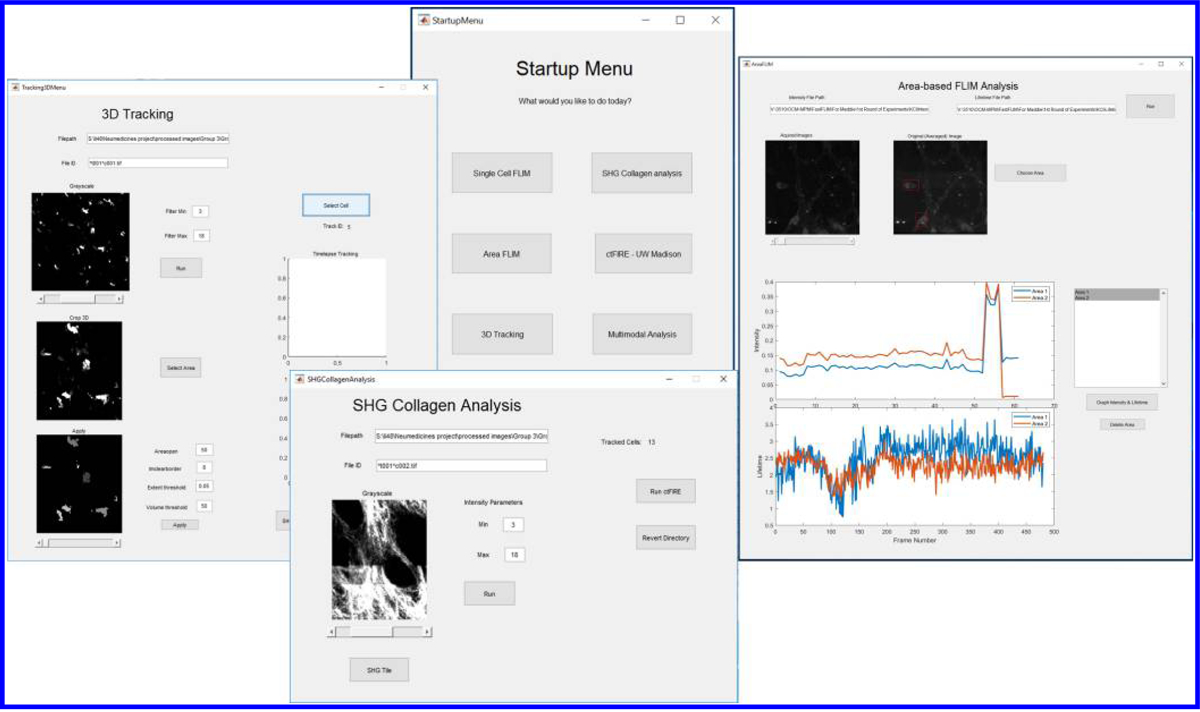
Schematic representation of the multimodal analysis platform. From the startup menu, users can select the analytical functions relevant to the study. The information collected from individual modules can be transferred to different analytical functions for cross-modality correlation.

The analysis platform was designed with Matlab Graphical User Interfaces (GUI), allowing users to control selected parameters to optimize both image segmentation and tracking outcome. The goal of creating this type of point-and-click control application is to provide an environment that can effectively utilize the unique and large amount of information offered by time-lapse *in vivo* multimodal multiphoton imaging. Figure 1 shows the layout of the analysis platform. The analysis platform can be broken down into three sections, which will be described in detail in the Results section.

### 2.2. Multiphoton microscopy

**Fig. 2.**
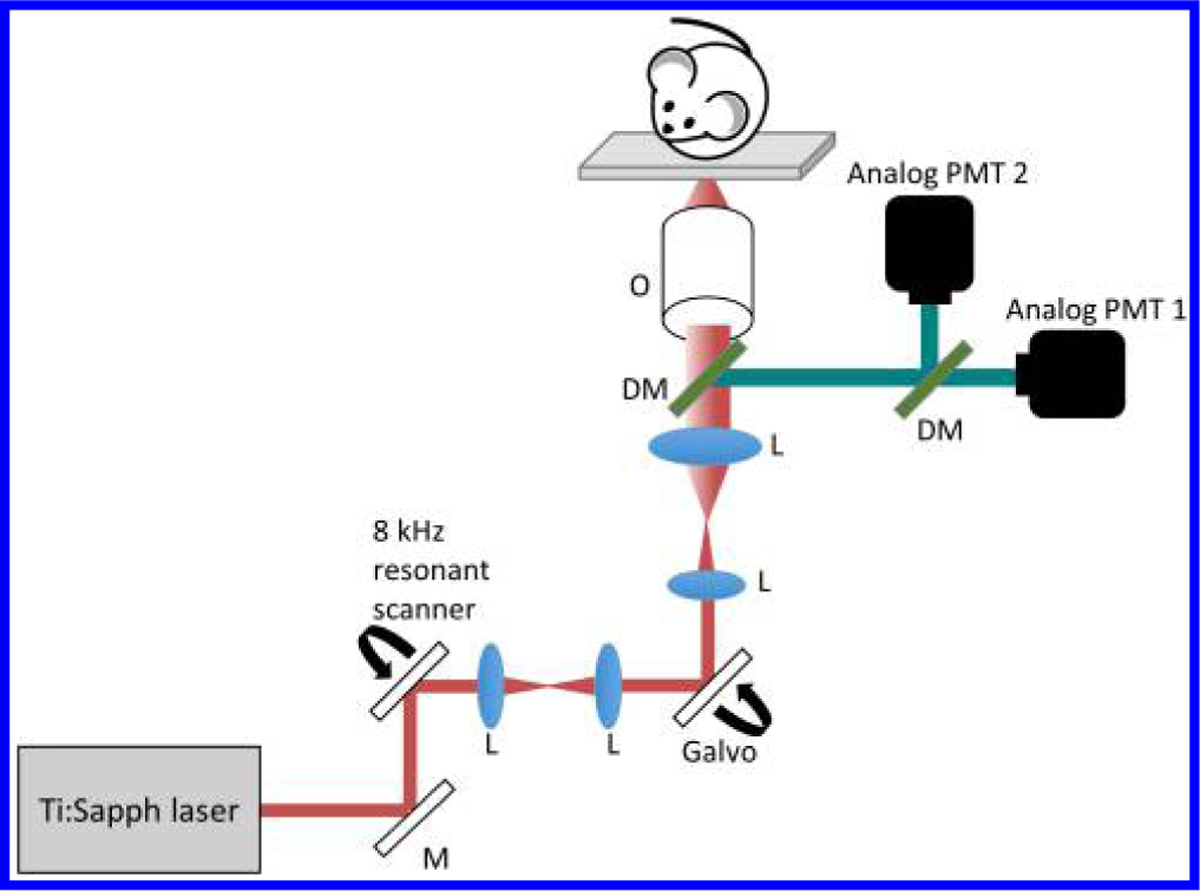
Schematic of the multimodal multiphoton microscope. M: mirror, L: lens, DM: dichroic mirror, O: objective.

All imaging was performed using a custom-built video-rate multimodal multiphoton microscope.^24^ With a faster scanning and detection rate, large FOV volumetric data of TPF, SHG, and FLIM images can be acquired in a spatially and temporally co-registered manner. Most importantly, the imaging acquisition time is significantly shorter by incorporating an 8 kHz resonant scanning mirror (EOPC SC-30) for the fast axis,^24^ allowing comparably larger FOV time-lapse images to be captured, compared to a previous custom-built system.^22,25,26^ In addition, the signals from the photomultiplier tubes (PMTs) are sent through a high bandwidth transimpedance amplifier (Hama-matsu C5594 – 1.5 GHz bandwidth) and directly digitized using a 12 bit, 1.8 gigasamples per second digitizer (AlazarTech ATS-9360).^24^ The laser excitation is provided by a mode-locked Ti:Sapphire laser (Spectra-Physics Mai Tai HP). TPF and SHG imaging was performed using 920 nm excitation wavelength, and FLIM images were acquired using 750 nm excitation wavelength. Figure 2 shows the schematic representation of the multimodal multiphoton microscope.

An inverted microscope design was implemented, with a removable glass coverslip positioned at the center of a motorized stage in between the imaging sample and the objective (Fig. 2). Utilizing the above stage design, the microscope is more versatile and can accommodate a wide range of imaging conditions, including *in vivo* animal, *in vitro* cell^27^ and human skin imaging.

### 2.3. In vivo multimodal volumetric imaging

This study was designed to focus on characterizing immune cell dynamics because this group of cells is essential in wound healing.^22,25,26^ The animals used in this representative study were four one-year-old female MacGreen B6N.Cg-Tg(Csflr-EGFP)Hume/J transgenic mice (Jackson Laboratory), and all animal procedures were performed under a protocol approved by the Institutional Animal Care and Use Committee (IACUC) at the University of Illinois at Urbana-Champaign. These mice express enhanced GFP (EGFP) in peritoneal, bone-marrow-derived, and broncho/alveolar macrophages, as well as in Langerhans cells. Unlike other GFP-expressing transgenic mouse models, the MacGreen model expresses GFP specifically in macrophages, and therefore is an ideal model for studying the *in vivo* dynamics of these immune cells. To prepare animals for imaging, the hair on the dorsal skin was carefully removed with an electric shaver and surgical tweezers under general anesthesia (1.3% isofluorane, 1.3% oxygen) to reduce background auto-fluorescence during imaging. To wound the skin while the animal was still under anesthesia, the shaved area was first cleaned with rubbing alcohol, after which a full-thickness skin wound was created using a sterile 1 mm biopsy punch (Miltex, Inc.), followed by additional cleaning with rubbing alcohol. The wounds were left uncovered, and no analgesic compounds were prescribed during the study, as these would potentially interfere with the *in vivo* immune response.

Time-lapse imaging was performed on the day of wounding (Day 1) and 7 days post-wounding (Day 7). On each imaging day, a 60 min TPF-SHG time-lapse image sequence was captured over a 6 × 6 × 5-frame volumetric mosaic with a 20 frame average per location and a 5 min time gap. During volumetric imaging, the motorized translational stage moved at a 150 *μ*m lateral step size and a 5 *μ*m axial step size. A 5 min time gap between volumetric mosaics was used to limit laser exposure and potential heating or injury from the continuous scanning laser exposure. A shorter time gap was not possible due to the time required to save the multimodal image data to the hard disk. Although a shorter time gap would be more ideal for capturing cell motility, one of the objectives of this study was also to perform large FOV analysis. Therefore, a large imaging volume with a slightly longer time gap was chosen empirically. It is important to mention that the 5 min time gap did not affect the 3D cell tracking significantly. This volumetric mosaic covered a region of approximately 1000 × 1000 × 20 *μ*m^3^. This volume of image data was able to capture a significant amount of cellular activity compared to our previous custom-built system.^22,25,26^ In addition, 6 × 6 FLIM mosaics with 80-frame average per stage location were acquired following the 60 min TPF-SHG time-lapse imaging, covering the same lateral dimensions as the TPF-SHG imaging to ensure spatially co-registered TPF-SHG-FLIM.

## 3. Results and Discussions

### 3.1. Development of cellular dynamics analysis parameters

The analysis platform includes three sections, which are described below.

#### 3.1.1. 3D cell tracking

One of the key aspects of this platform is 3D tracking of cell motility. This function is crucial because the *in vivo* environment is highly dynamic, and 2D tracking is often not sufficient to fully quantify cellular activity. In addition, breathing motion from the animals can minutely change the imaging depth during time-lapse imaging, and 3D tracking functionality allows observers to follow single-cell movement as accurately as possible. The first component of this module involves displaying and optimizing the volumetric grayscale images of the TPF cell data. Once the images are optimized, the user can crop the original image to isolate an area-of-interest for further analysis. The *x*–*y* coordinates of the cropped location can then be stored and utilized across different imaging modalities to ensure all subsequent analysis is performed at the same location where cells are tracked across all time points. Based on the newly selected area-of-interest, threshold-based cell segmentation is performed, which is followed by identifying detected cells, assigning all with numerical tags, and recording all corresponding 3D centroid coordinates. These locations serve as the initial time points for the time-lapse tracking.

To perform time-lapse tracking, the module allows the user to select an individual cell to be tracked. Using the recorded 3D centroid coordinates, the Euclidean distances between the cell at the current time point (t1) and all identified cells at the next time point (t2) are calculated. The cell with the smallest Euclidean distance was identified as the cell-of-interest at t2. This procedure was repeated between all following adjacent time points until the final time point indicated by the user. Initial characterization showed that the segmentation module can successfully separate individual cells if the distance between cells is visually separable. We also found the tracking method described here can track most cells reliably over time. Though it is possible that other cells can be mistakenly assigned as the t2 location of the cell-of-interest in high-density areas, this concern can be resolved by using smaller time-gap and axial sampling size during imaging. When completed, this module recorded the 3D centroid locations of all selected cells at all tracked time points. With the above information, the instantaneous velocity of all tracked cells can be determined. In addition, users can utilize these coordinates to perform a variety of analyses, such as the traveling direction of the selected cells. When combining travel direction with other data sources, such as collagen structure and fluorescence lifetime, the correlation among these data can be determined.

#### 3.1.2. Volumetric collagen structure characterization

The second part of the analysis module involves analysis of the SHG signal for collagen structure characterization. The goal is to design a module for this platform that enables collagen structure characterization at regions where tracked cells are located, as identified previously in the cell tracking module. Although collagen is a 3D structure, all analyses done in this module were performed in 2D due to the lower axial resolution of the multimodal microscope compared to the transverse resolution.

**Fig. 3.**
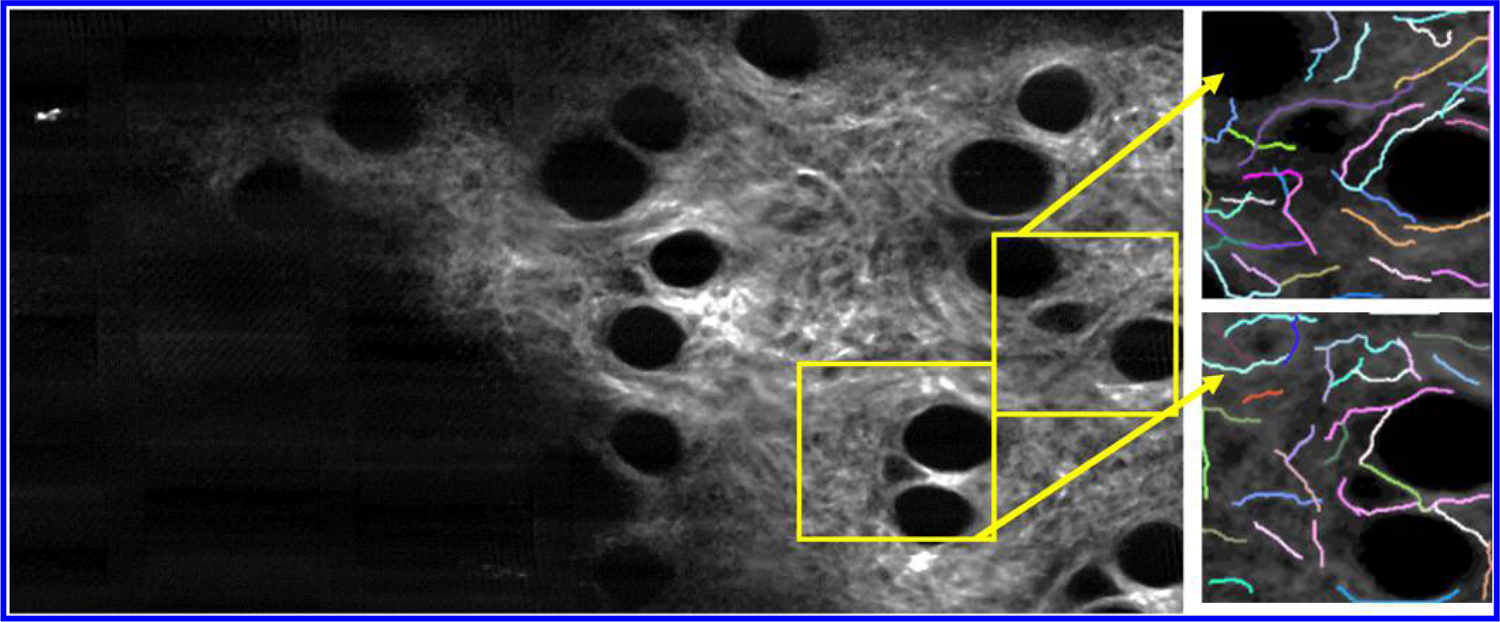
Cell-based regional SHG image tiles created by the collagen analysis module and analyzed using CT-FIRE. Selected individual collagen fibers are identified and the associated orientation angles can be calculated. The blank region at the lower left corner in the left image is associated with the inside of the skin wound.

This module first allows the user to optimize the image contrast in a manner similar to the cell tracking module. This function then automatically crops the original volumetric SHG image stack according to the cropping parameters recorded previously in the cell tracking module. The SHG region-of-interest image stack is further cropped into smaller SHG regions based on the locations of the tracked cell for the subsequent CT-FIRE analysis. Because the multimodal data is 3D, it is crucial that each 2D SHG collagen image be retrieved from the same depth as the tracked cell. To achieve this, a 200 by 200-pixel SHG image tile was created and centered around the associated tracked cell based on the 3D cell centroid coordinates. By utilizing the *z*-direction of the centroid coordinates, SHG analysis could be performed at the same depth as the tracked cell, as shown in Fig. 3. After sub-region SHG image tiles are created, the users can then execute the CT-FIRE collagen analysis tool linked to this module on each of the created image tiles.^13,17,18^ Through CT-FIRE, selected individual collage fibers can be identified, and the orientation angle of each collagen fiber can be calculated, as illustrated in Fig. 3.

An additional function in this module was the Fourier analysis of the same cell-based regional SHG image tiles.^14,28^ Two-dimensional spatial Fourier transforms (FT) are performed on these images, and the FT magnitude images are converted to binary images based on a specified threshold. An ellipse-fit is then performed on these binary images utilizing the Matlab Imaging Processing Toolbox function. To quantify the collagen structure and alignment around tracked cells, the eccentricity of each ellipse is used, defined as the ratio of the distance between the foci of the ellipse and its major axis length, and resulting in a value between 0 and 1. A value closer to 0 suggests that the collagen fibers in the selected regions are isotropically distributed, and a value closer to 1 suggests that the collagen fibers are more aligned in a particular direction.^28^ With the collagen parameters collected in this module, one can correlate them back to cell parameters and potentially quantify the relationship between collagen structure and cell motility.

#### 3.1.3. Area-based lifetime analysis

The last module is designed to perform area-based fluorescence lifetime analysis of intracellular reduced nicotinamide adenine dinucleotide (NADH) on large FOV FLIM images. NADH is an important coenzyme involved in the reduction-oxidation reaction in cells, and FLIM has been widely used to probe the metabolic activity in biological tissues.^23,24,29–31^ To effectively utilize the large FOV FLIM images, this module performed overall average NADH lifetime calculations as well as regional area-based NADH lifetime calculations.

In this module, both the NADH intensity and lifetime data are imported. As FLIM data was only acquired at a single depth in the epidermal layer of the skin, only 2D analysis was performed. Two types of lifetime calculations can be performed. The first utilizes the region-of-interest cropping information stored from the cell tracking module and calculates the average NADH lifetime across this region. The other method allows the users to select any area in the entire image and calculate the regional average lifetime. The incorporation of area-based analysis provides the users freedom to analyze areas outside the TPF-SHG analysis region. This information can provide evidence of the relationships among regional metabolic state, cell motility, and collagen structure. By combining these multimodal parameters quantified in both a temporally and spatially co-registered manner, additional information may be obtained regarding the biological conditions of living tissue, and the cellular activity can be studied in the native tissue microenvironment.

#### 3.1.4. Characterization of in vivo cellular dynamics

**Fig. 4.**
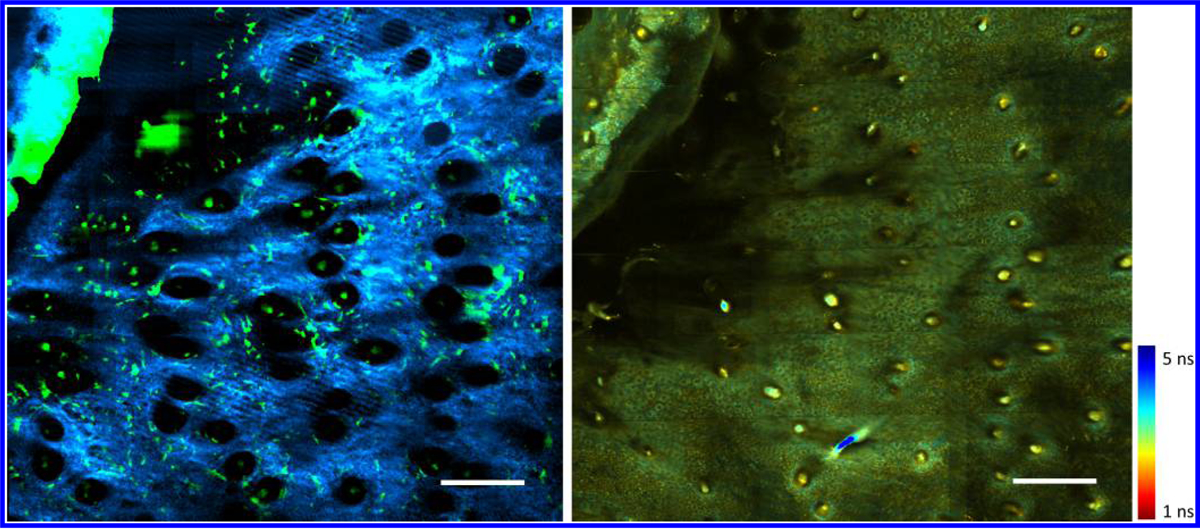
Temporally and spatially co-registered *in vivo* TPF-SHG-FLIM images. Left: TPF-SHG composite. Green represents GFP-labeled cells and blue represents collagen. Right: FLIM NADH lifetime image. Color bar: NADH lifetime. Scale bars: 200 *μ*m.

To demonstrate the utility of the multimodal analysis platform presented in this work, analysis was performed on multimodal multiphoton microscopy data obtained from a longitudinal wound healing study. The data collected included dynamics of macrophages captured through TPF, the associated collagen structure near the edge of the wound obtained through SHG imaging, and the functional metabolic tissue state through NADH lifetime imaging of the same region. Figure 4 illustrates representative co-registered TPF-SHG-FLIM images that demonstrate the vast amount of complementary information embedded in the data.

To the best of our knowledge, these datasets represent the first spatially co-registered TPF-SHG-FLIM images of living skin with such an extended FOV. A variety of the biological parameters were available for analysis in this dataset, including the relationship among cellular motility and orientation, collagen fiber alignment, and NADH lifetime.

**Fig. 5.**
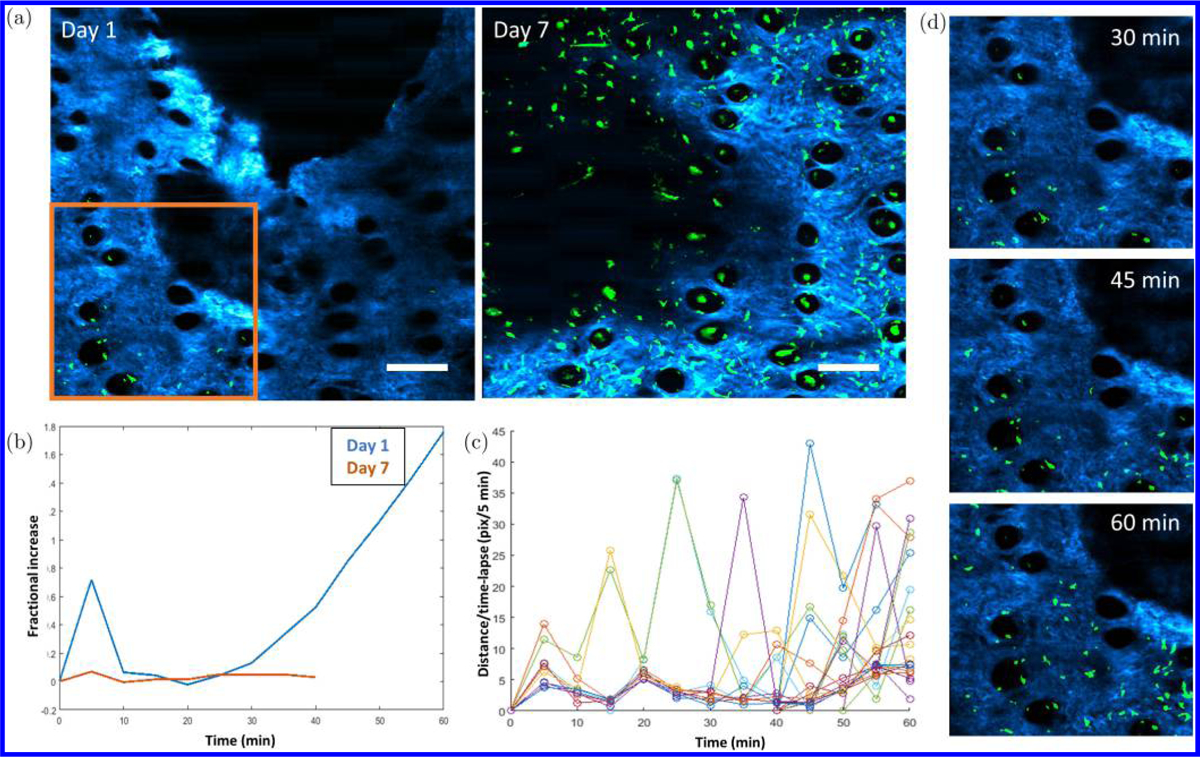
Longitudinally-tracked *in vivo* cell dynamics. (a) Cell mobility on Days 1 and 7. (b) A noticeable increase in cell influx toward the wound edge was observed on Day 1, as shown by the fractional increase of cells over time. (c) Instantaneous velocity over time shows a visual trend which resembles a contractile motion. (d) Day 1 time-lapse images from the location identified in (a) (orange box) shows the influx of cells over time. Scale bars: 200 *μ*m.

Using the image analysis platform presented above, noticeable influx of GFP cells toward the edge of wound is visualized on Day 1 after a wound was created. In addition, the corresponding FLIM image shows that the NADH lifetime around the edge of the wound appeared to be noticeably longer than the rest of the surrounding skin further away from the wound bed. In comparison, cellular motility on Day 7 is markedly decreased. These observations are presented in Fig. 5.

In addition, the change in cell population over time was calculated using the information provided by the 3D cell tracking. At Day 1, a noticeable increase in cell population is observed over the time duration of the experiment, which is consistent with visual observation (Fig. 5(d)). The instantaneous velocity of the time-lapse data was also calculated utilizing the time-lapse centroid locations of tracked cells at every time point (Fig. 5(c)). The plot shows a trend visually similar to a contractile motion, agreeing with previous studies.^1^ This observation can potentially be a visual indication of cells traveling along connective tissues, but more studies are needed to verify the current hypothesis.

**Fig. 6.**
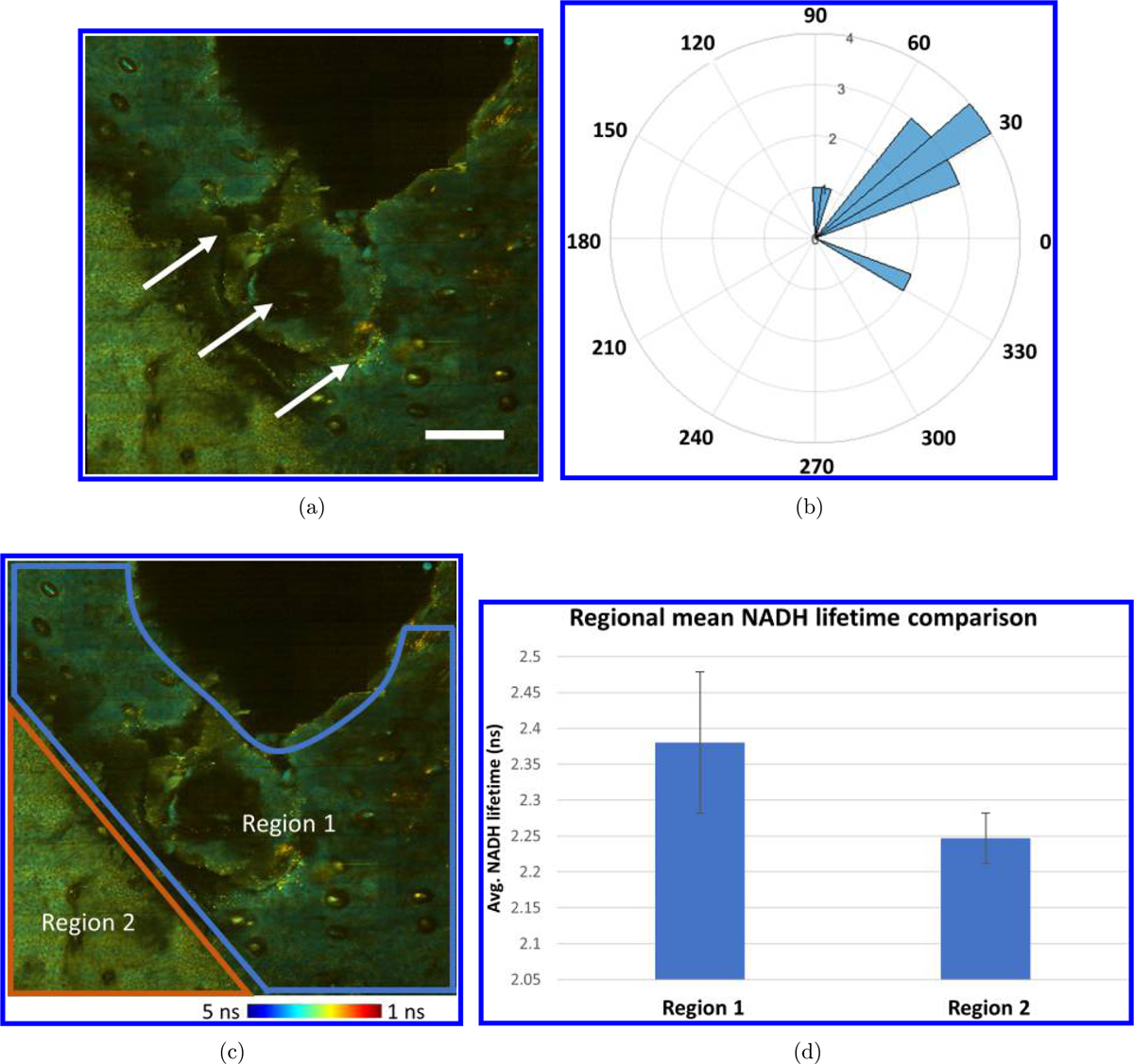
Cell migration and area-based FLIM analysis on Day 1 following wounding. (a) White arrows shown on large FOV FLIM image to illustrate the direction of cell migration. (b) Cell migration polar histogram shows most moving cells migrated along the direction shown in (a). (c) Additional area-based FLIM analysis illustrated that skin near the edge of wound (blue boundary, Region 1) appeared to have a longer mean NADH lifetime than the area more distant to the wound (orange boundary, Region 2). (d) Area-based NADH lifetime calculation confirms the mean lifetime in Region 1 is longer than that in Region 2. Color bar: NADH lifetime. Scale bar: 200 *μ*m.

To characterize the migration of cells toward the edge of the wound, the 3D cell tracking module was utilized. The initial and final centroid locations of each tracked cell were utilized to calculate the total displacement, and the inverse tangent was utilized to calculate the direction of cell migration. Analysis of cell migration on Day 1 reveals that most cells moved toward the direction of the edge of wound. The relationship between cell migration and location of wound is clearly shown in Fig. 6. Interestingly, a change in NADH lifetime can also be seen around the edge of wound (Fig. 6). To quantify this, area-based FLIM analysis was utilized by first randomly selecting five small areas from Region 1, and five areas from Region 2, as labeled in Fig. 6(c). The mean NADH lifetime of these regions was calculated by averaging the mean NADH lifetime of the five smaller areas in these regions. Results show a decreased lifetime in regions more distant from the wound (Region 2) compared to those surround the wound edge (Region 1).

Interestingly, increased cell migration was observed toward the wound edge, corresponding to these areas of longer NADH fluorescence lifetime. This observation can be best visualized by directly comparing cell migration data in Fig. 5(d) with area-based FLIM analysis in Fig. 6. The multimodal analysis presented here is unique and represents a powerful approach to study the *in vivo* dynamic microenvironment of wounded skin due to the combination of this multimodal image analysis platform and video-rate imaging system, allowing cellular-level analysis over large spatial scales *in vivo*. The results here show information in the micro- and macroenvironment that can potentially allow users to connect *in vivo* cell motility to functional and structural tissue parameters.

In addition to cell migration relative to NADH lifetime distribution, another biological parameter investigated was cell motility relative to collagen fiber angle. By taking advantage of the spatially co-registered multimodal data collection and analysis, local collagen structures of all tracked cells can be quantified using CT-FIRE in the SHG collagen analysis module. Combining with the time-lapse 3D locations of the tracked cells, users can determine relationships between *in vivo* cell motility and local connective tissue structures. However, no clear relationship between cell motility and collagen fiber structures were observed here due to the short time span of the study.

## 4. Conclusions

The results presented here demonstrate an integrated multimodal analysis platform to investigate *in vivo* biological parameters and phenomena that are challenging to characterize with traditional imaging and analysis techniques. While only a few cellular parameters and measurements are presented here to demonstrate both the importance of large FOV multimodal imaging and 3D characterization, many more can potentially be studied using this multimodal analysis platform. In addition, the study presented here is only a demonstration of the application of both the video-rate multimodal microscopy and the analysis module developed in this study. Additional experiments are needed to evaluate this analysis platform under different experimental conditions. Also, future *in vivo* studies should be performed on imaging volumes that can better represent the 3D regions, including higher sampling of the *z* dimension. Furthermore, the imaging volume in future time-lapse cell tracking should cover a larger axial dimension without losing significant amount of lateral dimension and the time-lapse information.

The multimodal analysis platform can be improved by including additional imaging modalities and analysis modules to address even more biological parameters. The design of this analysis platform is versatile, and the addition of other imaging modalities and parameters can potentially expand the applicability of this analysis platform to other biological phenomena, including the tumor microenvironment and neural activities.^2,9,13,17^ With the techniques presented here, the analysis of *in vivo* cellular dynamics can be transformed. By observing and quantifying biological phenomena in this manner, researchers may gain additional insights leading to a better understanding of specific biological microenvironments in health and disease.
